# Targeted next-generation sequencing of head and neck squamous cell carcinoma identifies novel genetic alterations in HPV+ and HPV- tumors

**DOI:** 10.1186/gm453

**Published:** 2013-05-29

**Authors:** Matthias Lechner, Garrett M Frampton, Tim Fenton, Andrew Feber, Gary Palmer, Amrita Jay, Nischalan Pillay, Martin Forster, Maureen T Cronin, Doron Lipson, Vincent A Miller, Timothy A Brennan, Stephen Henderson, Francis Vaz, Paul O'Flynn, Nicholas Kalavrezos, Roman Yelensky, Stephan Beck, Philip J Stephens, Chris Boshoff

**Affiliations:** 1 UCL Cancer Institute, University College London, 72 Huntley Street, London, WC1E 6BT, UK; 2 Head and Neck Centre, University College London Hospitals NHS Trust, Euston Road, London, NW1 2PG, UK; 3 Foundation Medicine, One Kendall Square, Suite B3501, Cambridge, MA 02139, USA; 4 Department of Histopathology, University College London Hospitals NHS Trust, Rockefeller Building, University Street, London, WC1E 6JJ, UK

## Abstract

**Background:**

Human papillomavirus positive (HPV+) head and neck squamous cell carcinoma (HNSCC) is an emerging disease, representing a distinct clinical and epidemiological entity. Understanding the genetic basis of this specific subtype of cancer could allow therapeutic targeting of affected pathways for a stratified medicine approach.

**Methods:**

Twenty HPV+ and 20 HPV- laser-capture microdissected oropharyngeal carcinomas were used for paired-end sequencing of hybrid-captured DNA, targeting 3,230 exons in 182 genes often mutated in cancer. Copy number alteration (CNA) profiling, Sequenom MassArray sequencing and immunohistochemistry were used to further validate findings.

**Results:**

HPV+ and HPV- oropharyngeal carcinomas cluster into two distinct subgroups. *TP53 *mutations are detected in 100% of HPV negative cases and abrogation of the G1/S checkpoint by *CDKN2A/B *deletion and/or *CCND1 *amplification occurs in the majority of HPV- tumors.

**Conclusion:**

These findings strongly support a causal role for HPV, acting via p53 and RB pathway inhibition, in the pathogenesis of a subset of oropharyngeal cancers and suggest that studies of CDK inhibitors in HPV- disease may be warranted. Mutation and copy number alteration of PI3 kinase (PI3K) pathway components appears particularly prevalent in HPV+ tumors and assessment of these alterations may aid in the interpretation of current clinical trials of PI3K, AKT, and mTOR inhibitors in HNSCC.

## Background

Human papillomavirus-related (HPV+) head and neck squamous cell carcinoma (HNSCC) is a subgroup of HNSCC where the incidence is increasing in most developed countries [1]. The vast majority of HPV+ HNSCC originate from the oropharynx, and in particular the tonsillar beds [2]. These tumors are almost exclusively associated with HPV-16, have integrated and functionally active E6 and E7 viral oncoproteins, and compared to HPV-negative tumors appear to have an overall better outcome, independent of treatment modality [3].

Whole-exome sequence analysis was previously performed to reveal the mutational landscape of HNSCC [4,5]. These studies showed that >80% of tumors contain *TP53 *mutations and strikingly up to 20% have loss-of-function *NOTCH1 *mutations. However, in these two studies, only seven and four HPV+ samples were included, respectively. Both studies confirmed the lack of *TP53 *mutations compared to HPV- samples, and overall, a lower mutational burden in HPV+ disease.

To further understand the contribution of somatic genomic alteration in the pathogenesis of HPV+ HNSCC we employed paired-end sequencing of hybrid-captured DNA, targeting 3,230 exons in 182 of the most common cancer-altered genes, plus 37 introns from 14 genes often rearranged in cancer.

## Methods

### Sample collection, p16 staining, and DNA extraction

Ethical approval for this study was granted by the UCL/UCLH Ethics Committee (Reference number 04/Q0505/59) with informed consent obtained where required. Based on the results of a power analysis and taking into account gender and age-matching requirements we selected 20 HPV+ and 20 HPV- oropharyngeal carcinomas (from 22 HPV+ and 34 HPV- oropharyngeal cancer samples available to us), all formalin fixed paraffin-embedded (Table 1). Our power analysis suggested that by choosing the described number of samples there was a just under 90% chance of detecting moderate differences in the proportion of mutations between HPV+ and HPV- HNSCC samples (w = 0.5, *P *= 0.05).

**Table 1:** Patient characteristics of selected HPV+ and HPV- HNSCC samples.

	HPV+ (*n *= 20)	HPV- (*n *= 20)
**Median age, years (range)**	56.5 (42-81)	58 (45-77)

**Gender**	M: 14	M: 14
	F: 6	F: 6

**Tumor site**	Oropharynx: 20	Oropharynx: 20

**Tumor grade**	Well diff: 1	Well diff: 0
	Mod diff: 9	Mod diff: 16
	Poorly diff: 10	Poorly diff: 4

**Tumor stage (T)**	T1: 5	T1: 1
	T2: 8	T2: 4
	T3: 3	T3: 5
	T4: 3	T4: 10
	N/a: 1	N/a: 0

**Cervical lymph node involvement (N)**	Yes: 16	Yes:13
	No: 2	No: 6
	N/a: 2	N/a: 1

**Smoking**	Ever: 9	Ever: 15
	Never: 8	Never: 0
	N/a: 3	N/a: 5

**Alcohol**	Heavy drinker (>20U/w): 2	Heavy drinker (>20U/w): 12
	Occ. alcohol: 5	Occ. alcohol: 3
	Never: 4	Never: 0
	N/a: 9	N/a: 5

**Figure 1:**
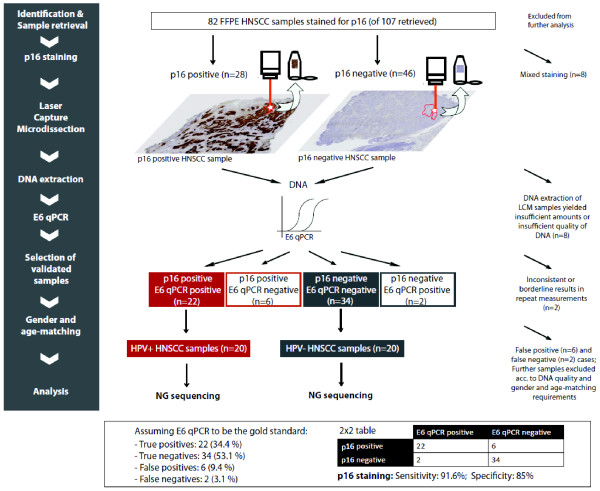
**Workflow of FFPE sample preparation and selection**. Eighty-two FFPE blocks [19] were stained for p16 of which eight samples were excluded from further analysis, showing mixed p16 staining. Eight samples were excluded after the LCM step, yielding insufficient amounts or quality of DNA and two further samples were excluded due to inconsistent or borderline results in repeat E6 qPCR measurements. In total, 22 confirmed HPV+ (p16+ and E6 qPCR+) and 34 HPV- (p16- and E6 qPCR-) samples were suitable for further analysis. Following age and gender matching, 20 HPV+ HNSCC samples (red) and 20 HPV- HNSCC samples (grey) were then selected for the final analysis (next-generation (NG) sequencing).

Details of sample preparation and selection are illustrated in Figure 1. We confirmed HPV status by p16 staining, and by quantitative PCR for HPV-16 E6, having been shown to have 97% sensitivity, 94% specificity, and to be the best discriminator of favorable outcome [6]. Sequencing of HPV DNA demonstrated 100% concordance of HPV status. All samples were laser-capture microdissected (LCM) to separate tumor epithelial from surrounding stromal tissues, enriching tumor DNA for further analyses. These were processed as 10 μm thick unstained slides which were reviewed by an expert pathologist who had marked the slides for tumor subtype enrichment in a corresponding H&E stained section. LCM was carried out on P.A.L.M. MembraneSlide 1.0 PEN slides (*Zeiss *Microimaging, Munich, Germany) using the Zeiss Palm MicrobeamTM system. Tissue was collected into extraction tubes and processed using the QIAamp DNA FFPE Tissue Kit (Qiagen, Hilden, Germany). Extracted DNA was quantified using a standardized PicoGreen fluorescence assay (LifeTechnologies, Carlsbad, CA, USA).

### DNA library construction and hybrid capture

At least 50 ng and up to 200 ng of extracted DNA was sheared to approximately 100-400 bp by sonication, followed by end-repair, dA-addition and ligation of indexed, Illumina sequencing adaptors. Sequencing libraries were hybridization captured using RNA-based baits (Agilent), targeting a total of 3,320 exons of 182 cancer-related genes (most commonly altered in cancer, from [7]) plus 37 introns from 14 genes often rearranged in cancer (Additional File 1, Table S1).

### Sequencing and primary sequence data analysis

Paired end sequencing (49 × 49 cycles) was performed using the HiSeq2000 (Illumina). Six samples yielded insufficient numbers of reads and were excluded from analysis. The summary of sequencing details is illustrated in Additional File 1, Table S2. Sequence data from gDNA, available from 18 HPV+ and 16 HPV- samples, were mapped to the reference human genome (hg19) using the BWA aligner [8]. PCR duplicate read removal and sequence metric collection was performed using Picard [9] and SAMtools [10]. Local alignment optimization was performed using GATK [11]. Hybrid capture reagents included baits designed to capture unique regions of select viral genomes including HPV-16. Sequence read pairs were aligned to the reference genome of the respective viral genomes, and the number of pairs mapping to each viral genome was counted. A total HPV-16 aligned read count of ≥5 reads per million was considered a positive HPV status, and ≤2 negative HPV status.

### Genomic alteration detection

Base substitution detection was performed using a Bayesian methodology, which allows detection of novel somatic mutations at low MAF and increased sensitivity for mutations at hotspot sites [12] through the incorporation of tissue-specific prior expectations: P(Mutationpresent|Readdata"R")=P(Frequencyofmutation"F">0|R)∝1-P(R|F=0)P(F=0), where P(R|F) is evaluated with a multinomial distribution of the observed allele counts using empirically observed error rates and P(F=0) is the prior expectation of mutation in the tumor type. To detect indels, *de-novo *local assembly in each targeted exon was performed using the de-Bruijn approach [13]. Candidate calls are filtered using a series of quality metrics, including strand bias, read location bias, and a custom database of sequencing artifacts derived from normal controls. Germline alterations are identified and filtered using dbSNP (version 135 [14]) and subsequently annotated for known and likely somatic mutations using the COSMIC database (version 62, http://cancer.sanger.ac.uk/cancergenome/projects/cosmic/). Detection of copy-number alterations (CNAs) was performed by obtaining a log-ratio profile of the sample by normalizing the sequence coverage obtained at all exons against a process-matched normal control. The profile is segmented and interpreted using allele frequencies of ~1,800 additional genome-wide SNPs to estimate tumor purity and copy number based on established methods [15-17] by fitting parameters of the equation $lr_{seg}\sim N\left({\text{log}}_{2}\frac{p*C_{seg}+\left(1-p\right)*2}{p*tumor\;ploidy+\left(1-p\right)*2}\right)$, where $lr_{seg}$, $C_{seg}$, and *p *are the log-ratios and copy numbers at each segment and sample purity respectively. Focal amplifications are called at segments with ≥6 copies and homozygous deletions at 0 copies, in samples with purity >20%.

A summary of known and likely somatic or functional base substitution and indel (short-variant) alterations and of base substitution and indel (short-variant) alterations of unknown status detected by deep sequencing is illustrated in Additional File 1, Table S3 and Additional File 1, Table S4, respectively. A summary of copy number alterations detected by deep sequencing is illustrated in Additional File 1, Table S5.

### Validation of selected mutations by Sequenom OncoCarta

DNA extracted from FFPE samples were sent to Sequenom (Hamburg, Germany) for blind testing and analysis, using Sequenom OncoCarta panels v1.0 and v3.0, as previously described [18].

### Confirmation of copy number changes by Infinium CNA profiling

Using previously obtained Infinium HumanMethylation450 BeadChip methylation data on sequenced samples [19], the Bioconductor package 'DNAcopy' [20,21] was applied to calculate the copy number of the majority of sequenced samples, as described previously [22]. All normalized and raw 450k methylation data were submitted to GEO (Gene Expression Omnibus, NCBI) according to instructions provided (GEO accession number: GSE38266).

### Immunohistochemistry and interpretation of results

The sequenced 18 HPV+ and 16 HPV- HNSCC samples were stained for PTEN and for Cyclin D1. Staining for these particular targets was chosen as these were already implicated in HNSCC carcinogenesis and validated scoring systems are available [23,24]. Antibody 04-409 (Millipore-Merck KGaA, Darmstadt, Germany) was used for PTEN staining and antibody P2D11F11 (Novocastra) was used Cyclin D1 staining of 10-μm thick slides. The stained slides were examined and scored as previously described [23,24] by two experienced histopathologists.

### Statistical data analysis

Significance of enrichment of observed genomic alterations in HPV+ and HPV- HNSCC cases was tested using Pearson's chi-squared test. Relation of gender, tumor site, tumor grade, size of primary tumors (T), lymph node metastasis (N), smoking status, and alcohol intake to the two tested groups was determined using the Wilcoxon rank sum test. Relation of age to the two groups was tested by a logistic regression model. The obtained *P *values were corrected for multiple testing (FDR adjustment). Correlation of sequencing results with CCND1 and PTEN immunochemistry was tested using Fisher's exact test.

## Results

### Patient demographic data

**Figure 2:**
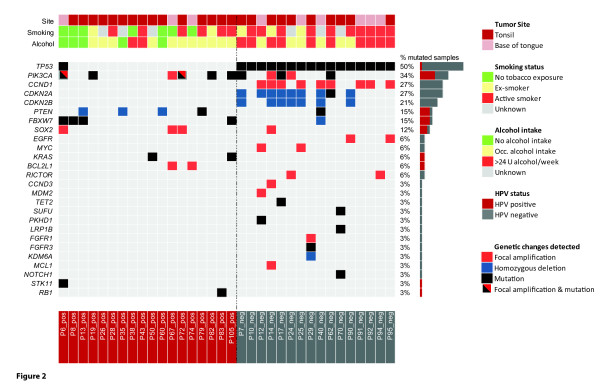
**Illustration of somatic events in HPV+ and HPV- HNSCC revealed by NGS of cancer-related genes**. Relevant demographic and histological data are described above the heatmap of genomic changes. The color coding of the observed changes and patient characteristics are explained in the key on the right.

The median age is slightly higher in the HPV- group (58 *vs*. 56.5 years) (Table 1). The male to female ratio is similar between the groups, and the majority of cases show moderately or poorly differentiated histology with evidence of lymph nodal involvement at presentation. In our cohort, as predicted, the vast majority of HPV- cases are in active smokers and/or heavy alcohol users (Table 1 and Figure 2). No significant relationship of gender, tumor site, tumor grade, size of primary tumors (T), lymph node metastasis (N), smoking status, determined using the Wilcoxon rank sum test, to any of the two tested groups (HPV+ HNSCC *vs*. HPV- HNSCC) was seen. Patients with high alcohol intake were significantly enriched in the HPV- group (Wilcoxon rank sum test; adjusted *P *value <0.05).

### Next-generation sequencing

**Figure 3:**
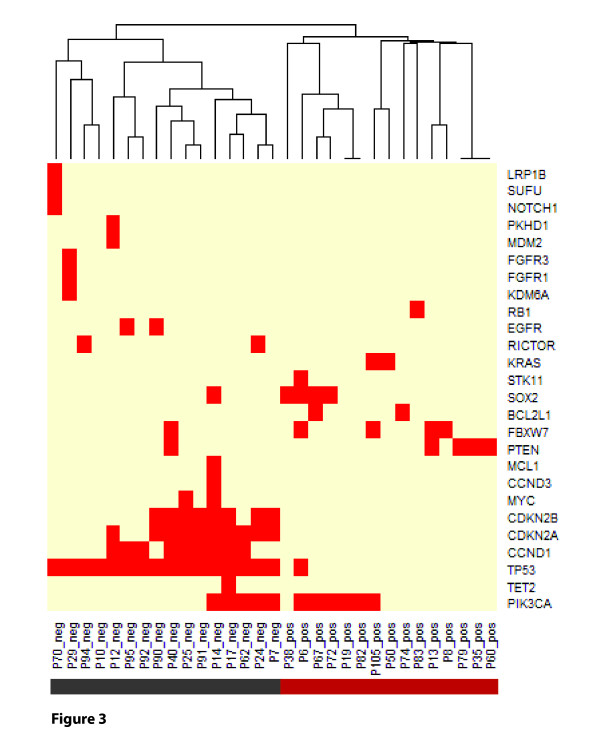
**Hierarchical clustering of HPV+ and HPV- HNSCC samples using all detected genetic changes**. HPV+ and HPV- HNSCC samples clustered in 100% of cases.

Sequence analysis revealed that HPV+ and HPV- oropharyngeal carcinomas cluster into two distinct subgroups, with few overlapping genetic alterations (Figures 2 and 3). *TP53 *mutations are detected in 100% of HPV- samples (Figure 2; significant enrichment in HPV- group; chi-square test, q <0.01). The list of observed *TP53 *mutations is illustrated in Additional File 1, Table S6. *CCND1 *amplifications (chi-square test, q <0.01) and *CDKN2A/B *deletions (chi-square test, q <0.05) were exclusively detected in HPV- cases (in approximately 55% and 40% of cases). *PIK3CA *mutation or amplification, and *PTEN *inactivation by gene copy loss or mutation were seen in >55% of HPV+ tumors, and in 31% HPV- tumors. *FBXW7 *alterations were present in >15% of all samples and *SOX2 *amplification in 12% of cases.

### Validation of obtained results

**Figure 4:**
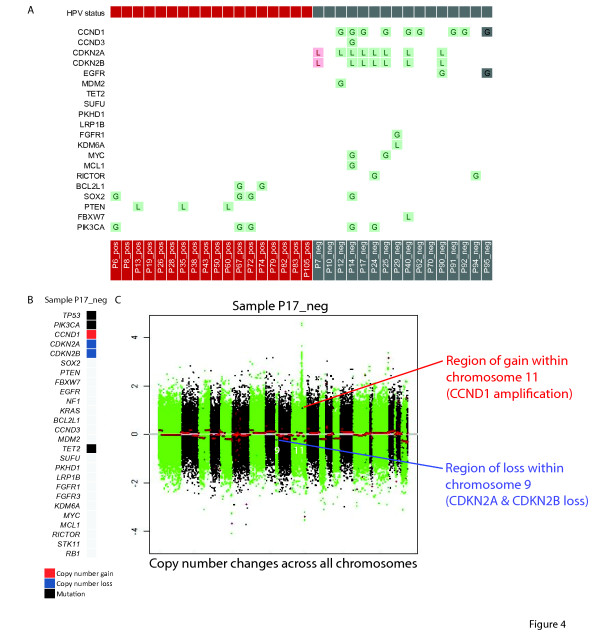
**Validation of copy number changes by Infinium CNA profiling across all samples**. (**A**) Forty-eight of 50 (96%) copy number alterations detected by sequencing were confirmed (green: confirmed, pink: not confirmed, grey: no data); (**B**) Genetic changes in 'P17_neg' detected by NGS (extracted from Figure 2); (**C**) Illustration of copy number changes (obtained from Infinium CNA Profiling) in 'P17_neg'. Both the loss of the *CDKN2A *and *CDKN2B *genes (in a region of loss within chromosome 9) and the gain of the *CCND1 *gene (in an amplified region of chromosome 11) are shown. Y-axis: log fold change of copy number, X-axis: copy number changes across all chromosomes.

For validation of our results, we applied Infinium CNA profiling, Sequenom OncoCarta panels v1.0 and v3.0 and immunohistochemistry. Copy number gains and losses detected by next-generation sequencing (NGS) were interrogated by Infinium CNA profiling (Additional File 2, Figure S1). Forty-eight of fifty (96%) copy number alterations detected by sequencing were confirmed (Figure 4). Furthermore, the detected mutations by NGS were validated by Sequenom OncoCarta panels v1.0 and v3.0 (Additional File 2, Figure S2). As our NGS technique targeted the whole gene sequence, whereas Sequenom OncoCarta panels only target specific mutational hotspots of certain genes, the majority of NGS detected mutations were not included in the Sequenom analysis. Eight out of nine mutations that were detected by NGS were also confirmed by Sequenom. One *PIK3CA *mutation in sample P72_pos was called at 1% allele frequency by NGS, and this mutation was therefore unlikely to be detected by Sequenom analysis.

**Figure 5:**
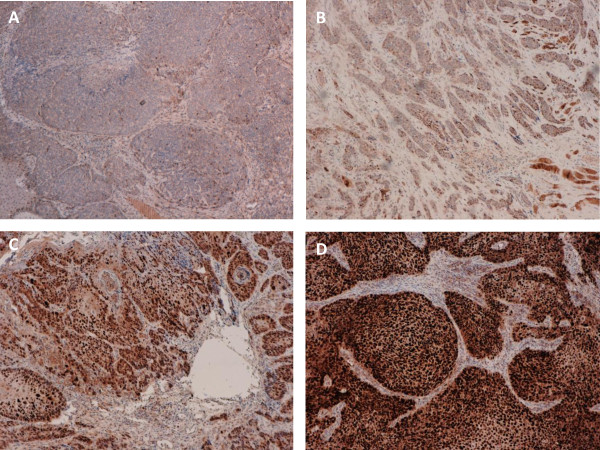
**Validation of detected copy number alterations of *Cyclin D1 (CCND1*) by immunohistochemistry**. Staining of HNSCC samples for Cyclin D1 confirmed strong expression in eight of nine *CCND1 *amplified cases (and intermediate expression in the remaining case) compared with samples harboring no copy number alteration; Representative samples shown: Low levels of *CCND1 *expression in the tumor tissue of sample 'P38_pos' (**A**) and sample 'P29_neg' (**B**); NGS: No CNA; High levels of Cyclin D1 expression in the tumor tissue of sample 'P12_neg' (**C**) and sample 'P17_neg' (**D**); NGS: *CCND1 *copy number gain.

**Figure 6:**
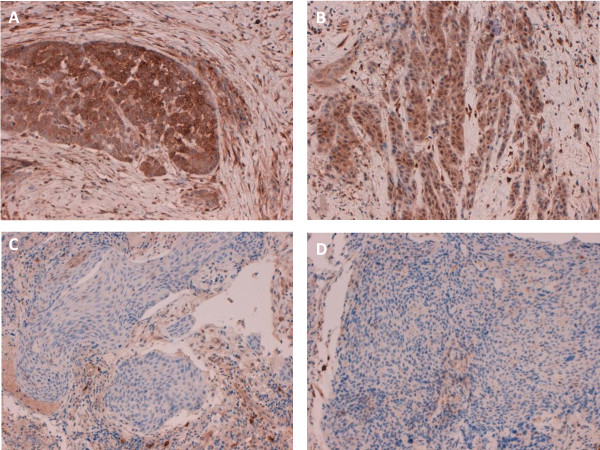
**Validation of detected *PTEN *copy number loss by immunohistochemistry**. Staining of HNSCC samples for PTEN was negative in all cases in which deep sequencing revealed a homozygous deletion or mutation. Representative samples shown: Abundant PTEN expression in the tumor tissue of sample 'P26_pos' (**A**) and sample 'P70_neg' (**B**); Deep-sequencing: No CNA; Absence of PTEN protein in the tumor tissue of sample 'P60_pos' (**C**) and sample 'P13_pos' (**D**); Deep-sequencing: *PTEN *copy number loss.

For *CCND1 *and *PTEN *we also validated findings by immunohistochemistry in sample material from the 18 HPV+ and 16 HPV- HNSCC samples tested by NGS. Genomic alterations in *CCND1 *were confirmed by Cyclin D1 immunochemistry with strong expression of Cyclin D1 protein in eight of nine *CCND1 *amplified cases (and intermediate expression in the remaining case). Using all tested samples, significant correlation of *CCND1 *sequencing results with Cyclin D1 immunochemistry was observed (*P *= 7.34e-05; Fisher's exact test). Representative samples are shown in Figure 5. *PTEN *loss and mutation were validated by immunohistochemistry (Figure 6). *PTEN *staining was negative in all cases in which NGS revealed a homozygous deletion or mutation. Four additional samples displayed low PTEN protein expression. In three of these cases a heterozygous deletion/single copy loss of *PTEN *was present, as detected by NGS. In the remaining sample other mechanisms may explain the loss of expression, such as an epigenetic alteration or changes in the post-transcriptional regulation of *PTEN*. Overall highly significant correlation of *PTEN *sequencing results with PTEN immunochemistry was demonstrated (*P *= 0.0009; Fisher's exact test).

Mutations reported in this study as 'known somatic' were limited to those that have been previously confirmed to be somatic in other tumors, through sequencing of matched normal specimens. Consequently, we are confident that these alterations are somatic.

## Discussion

Overall, sequence analysis revealed that HPV+ and HPV- oropharyngeal carcinomas cluster into two distinct subgroups, with few overlapping genetic alterations. These data concur with epidemiological and clinical data, indicating that HPV+ HNSCC is a distinct disease entity [25,26].

Our detection of *TP53 *mutations in 100% of HPV- samples, higher than previously reported [27], suggests that our approach of laser capture microdissection coupled with targeted deep sequencing is a highly sensitive method by which to assay specific tumor mutations. Taken together with the fact that in the HPV+ tumors, p53 function is suppressed by E6, our data suggest an obligate requirement for p53 abrogation in oropharyngeal tumorigenesis. One caveat in our study is that all HPV- samples analyzed were also p16 negative, thus it remains possible that in HPV- samples with elevated p16 expression (for example, through *RB1 *mutation), the frequency of *TP53 *mutation is <100%.

We identified only one *TP53 *mutation in an HPV+ tumor. However, this mutation (R290C, Additional File 1, Table S2) causes only a 40% decrease in *TP53 *function and has been detected in sarcomas harboring *MDM2 *amplification [28,29].

Our data for HPV- oropharyngeal cancer indicate that the frequency of *CCND1 *amplification (in approximately 55% of cases) and *CDKN2A/B *deletions (in approximately 55% of cases) are higher than previously reported [30]. *CCND1 *amplification has also been described in 12% of non-small cell lung cancers [31] and in up to 41% of esophageal squamous cell carcinomas [32], suggesting that this could be one of the more common genetic alterations linked to smoking-induced epithelial malignancy. In HPV+ cancer, the oncoprotein E7 leads to cell cycle dysregulation by substituting for cyclin D gain-of-function and cyclin dependent kinase inhibitor loss-of-function activities. Overall, this indicates that direct dysregulation of the cell cycle is a key mechanism for oropharyngeal tumors to evolve.

HPV+ HNSCC samples frequently harbor mutations or CNAs in genes implicated in activation of the PI3K/AKT/mTOR pathway. In particular, *PIK3CA *mutation and *PTEN *inactivation by gene copy loss or mutation were seen in >60% of HPV+ tumors, and in 31% HPV- tumors. There is a significant relation between PIK3CA and PTEN, and HPV status; chi-square test, *P *<0.001. These findings may help to explain the high frequency of PI3K pathway activation in HPV+ HNSCC samples and the efficacy of mTOR inhibitors in xenograft studies with HPV+ cell lines previously reported [33]. It will be important to audit both the sequence and copy number of the *PIK3CA *and *PTEN *genes if such agents are tested in clinical trials for HPV-associated HNSCC.

Our results suggest that mutations in *FBXW7 *may be enriched in HPV+ disease. FBXW7 is an E3 ubiquitin ligase that targets a number of growth-promoting proteins for proteasomal degradation, including Cyclin E, MYC, NOTCH and mTOR [34,35]. Loss of *FBXW7 *occurs in combination with *NOTCH *gain-of-function mutations in T-ALL [36], suggesting it may be an important target for FBXW7 ligase activity in these tumors. In contrast, HNSCC frequently display *NOTCH *loss-of-function-mutations [37,38], thus in HNSCC, other substrates such as Cyclin E, MYC, or mTOR may be the relevant targets for FBXW7. We found one HPV- sample harboring a *NOTCH1 *mutation, concurring with previous studies reporting *NOTCH1 *mutations in HNSCC [4,5].

Two of our tested HPV+ samples harbored *KRAS *mutations. *KRAS *mutations have been associated with a history of smoking [39]. One of the patients was a smoker and in the other one the smoking status was unknown. *HRAS *mutations were not detected in any of our tested samples. In previous studies, mutations in the *HRAS *gene were mainly detected in oral cavity cancer samples [4,5].

The *SOX2 *and *PIK3CA *genes both reside on the long arm of chromosome 3 (3q26) and these genes were amplified in three HPV+ samples and one HPV- tumor. While *PIK3CA *amplifications have previously been reported in HPV+ HNSCC [40,41], *SOX2 *has recently been proposed as the critical target of 3q gains observed at a high frequency in squamous lung cancer [42] and in esophageal squamous cell carcinoma [43]. *SOX2 *is also frequently amplified and overexpressed in oral squamous cell carcinoma [44]. Furthermore, *SOX2 *expression is upregulated in a subpopulation of putative HNSCC stem cells that displays characteristics of epithelial to mesenchymal transition (EMT), associated with increased propensity for metastasis [45].

We also demonstrate for the first time inactivating mutations in *STK11 *in HPV+ HNSCC. Loss of *STK11 *is associated with metastasis in head and neck cancer [46]. Furthermore, loss of function mutations in *STK11 (LKB1*) result in activation of mTORC1 signaling and can sensitize cells to mTOR inhibition [47,48]. Mutations in these genes therefore (in addition to *PIK3CA *and *PTEN*) warrant evaluation as potential determinants of sensitivity to mTOR inhibitors currently in clinical trials for HNSCC [49].

Beyond the genes directly involved in signaling and cell cycle, we found amplifications in genes implicated in preventing apoptosis: *BCL2L1 *(6% amplification) and *MCL1 *(3% amplification), suggesting that direct suppression of apoptosis may also contribute to HNSCC pathogenesis.

Receptor tyrosine kinase mutations, *FGFR1, FGFR3*, and *EGFR*, were only observed in HPV- tumors at low frequency.

Overall, our data strongly support a causal role for HPV in oropharyngeal carcinogenesis by overcoming the requirement for genetic lesions in the TP53 and RB1 tumor suppressor pathways evident in the HPV- tumors. Our detection of frequent PI3K/AKT/mTOR pathway alterations in HPV+ tumors is consistent with a recent report demonstrating PI3K pathway activation and sensitivity to mTOR inhibition in both cervical carcinoma and HPV+ HNSCC [33]. Together, these studies provide a rationale for the testing of PI3K pathway inhibitors in HPV+ HNSCC. In HPV- tumors, the frequent alteration of *CDKN2A/B *and/or *CCND1 *suggests that, if supported by functional data, trials with CDK inhibitors may be indicated. Our data support the observations by gene expression microarrays and by genome-wide methylation studies that HPV+ HNSCC is a distinct entity, with a distinct set of somatic alterations. However, it would appear that a core set of pathways (TP53, RB1/cell cycle, and PI3K/AKT/mTOR) is compromised in both HPV+ and HPV- oropharyngeal tumors, thus targeted therapies directed against one or more of these pathways could be efficacious in both contexts.

## Abbreviations

CNA: Copy number alteration; EMT: epithelial to mesenchymal transition; FF: fresh-frozen; FFPE: formalin-fixed paraffin-embedded; GEO: Gene Expression Omnibus; HNSCC: head and neck squamous cell cancer; HPV: human papillomavirus; HPV+: HPV positive; HPV-: HPV negative; LCM: laser-capture microdissected; NGS: next-generation sequencing; PI3K: PI3 kinase.

## Competing interests

NGS of samples was in collaboration with Foundation Medicine.

Employees of Foundation Medicine: GF, MTC, DL, VAM, RY, PJS

## Authors' contributions

All authors contributed to the interpretation of data and to the writing of the manuscript. In detail: Study design and conceptualization of study: ML, GF, TF, GP, SB, CB; Sample preparation, tumor collection, and technical work: ML, GF, TF, MF, FV, PO'F, NK; Histology and Computational Biology: AJ, AF, TAB, RY, NP, PJS; Figures and Tables: ML, GF, TF, AF. All authors read and approved the final manuscript.

## Supplementary Material

Additional file 1 **Table S1: 182 genes sequenced across entire coding sequence (A) and 14 genes sequenced across selected introns (B)**. Table S2: Summary of sequencing details for study samples. Table S3: Summary of known and likely somatic or functional base substitution and indel (short-variant) alterations detected by deep sequencing. Table S4: Summary of base substitution and indel (short-variant) alterations of unknown status detected by deep sequencing. Table S5: Summary of copy number alterations detected by deep sequencing. Table S6: List of *TP53 *mutations revealed by deep sequencing in HPV+ and HPV- HNSCC samples. Click here for file

Additional file 2 **Figure S1: Infinium CNA profiling of HPV+ and HPV- HNSCC samples**. Obtained genome-wide copy number alteration profiles (cumulative frequencies) between the two groups are illustrated and chromosomes displaying similar patterns of gain and loss in HPV+ and HPV- HNSCC samples are boxed. Chromosome 6 (MHC regions) and Y chromosome are not shown. Figure S2: Validation of detected mutations by SequenomOncoCarta panels v1.0 and v3.0. Mutations in HNSCC samples detected by deep sequencing were validated using the OncoCartapanels v1.0 and v3.0. 8 out of 9 mutations that were successfully tested on the Oncocarta panel were confirmed (green: confirmed, pink: not confirmed, grey: n/a). *The *PIK3CA*_E545K mutation in sample P72_pos was called at 1% allele frequency by NGS, and this mutation was therefore unlikely to be detected by Sequenom analysis. Click here for file
