# Supplementary material for: Targeted next-generation sequencing of head and neck squamous cell carcinoma identifies novel genetic alterations in HPV+ and HPV- tumors
Source: Genome Med. 2013 May 29;5(5):49. doi: 10.1186/gm453 (PMC4064312; doi:10.1186/gm453)
Supplement: Additional file 1 — Table S1: 182 genes sequenced across entire coding sequence (A) and 14 genes sequenced across selected introns (B). Table S2: Summary of sequencing details for study samples. Table S3: Summary of known and likely somatic or functional base substitution and indel (short-variant) alterations detected by deep sequencing. Table S4: Summary of base substitution and indel (short-variant) alterations of unknown status detected by deep sequencing. Table S5: Summary of copy number alterations detected by deep sequencing. Table S6: List of TP53 mutations revealed by deep sequencing in HPV+ and HPV- HNSCC samples. [file gm453-S1.DOCX]

| **Gene name**  **A** | | | | | **B** | **Gene name** |
| --- | --- | --- | --- | --- | --- | --- |
| *ABL1* | *CDK6* | *FLT4* | *MEN1* | *PTPN11* |  | *ALK* |
| *ABL2* | *CDK8* | *FOXP4* | *MET* | *PTPRD* |  | *BCR* |
| *AKT1* | *CDKN2A* | *GATA1* | *MITF* | *RAF1* |  | *BRAF* |
| *AKT2* | *CDKN2B* | *GNA11* | *MLH1* | *RARA* |  | *EGFR* |
| *AKT3* | *CDKN2C* | *GNAQ* | *MLL* | *RB1* |  | *ETV1* |
| *ALK* | *CEBPA* | *GNAS* | *MPL* | *RET* |  | *ETV4* |
| *APC* | *CHEK1* | *GPR124* | *MRE11A* | *RICTOR* |  | *ETV5* |
| *AR* | *CHEK2* | *GUCY1A2* | *MSH2* | *RPTOR* |  | *ETV6* |
| *ARAF* | *CRKL* | *HOXA3* | *MSH6* | *RUNX1* |  | *EWSR1* |
| *ARFRP1* | *CRLF2* | *HRAS* | *MTOR* | *SMAD2* |  | *MLL* |
| *ARID1A* | *CTNNB1* | *HSP90AA1* | *MUTYH* | *SMAD3* |  | *RAF1* |
| *ATM* | *DDR2* | *IDH1* | *MYC* | *SMAD4* |  | *RARA* |
| *ATR* | *DNMT3A* | *IDH2* | *MYCL1* | *SMARCA4* |  | *RET* |
| *AURKA* | *DOT1L* | *IGF1R* | *MYCN* | *SMARCB1* |  | *TMPRSS2* |
| *AURKB* | *EGFR* | *IGF2R* | *NF1* | *SMO* |  |  |
| *BAP1* | *EPHA3* | *IKBKE* | *NF2* | *SOX10* |  |  |
| *BCL2* | *EPHA5* | *IKZF1* | *NKX2-1* | *SOX2* |  |  |
| *BCL2A1* | *EPHA6* | *INHBA* | *NOTCH1* | *SRC* |  |  |
| *BCL2L1* | *EPHA7* | *INSR* | *NPM1* | *STAT3* |  |  |
| *BCL2L2* | *EPHB1* | *IRS2* | *NRAS* | *STK11* |  |  |
| *BCL6* | *EPHB4* | *JAK1* | *NTRK1* | *SUFU* |  |  |
| *BRAF* | *EPHB6* | *JAK2* | *NTRK2* | *TBX22* |  |  |
| *BRCA1* | *ERBB2* | *JAK3* | *NTRK3* | *TET2* |  |  |
| *BRCA2* | *ERBB3* | *JUN* | *PAK3* | *TGFBR2* |  |  |
| *CARD11* | *ERBB4* | *KDM6A* | *PAX5* | *TNFAIP3* |  |  |
| *CBL* | *ERCC2* | *KDR* | *PDGFRA* | *TNKS* |  |  |
| *CCND1* | *ERG* | *KIT* | *PDGFRB* | *TNKS2* |  |  |
| *CCND2* | *ESR1* | *KRAS* | *PHLPP2* | *TOP1* |  |  |
| *CCND3* | *EZH2* | *LRP1B* | *PIK3CA* | *TP53* |  |  |
| *CCNE1* | *FANCA* | *LRP6* | *PIK3CG* | *TSC1* |  |  |
| *CD79A* | *FBXW7* | *LTK* | *PIK3R1* | *TSC2* |  |  |
| *CD79B* | *FGFR1* | *MAP2K1* | *PKHD1* | *USP9X* |  |  |
| *CDH1* | *FGFR2* | *MAP2K2* | *PLCG1* | *VHL* |  |  |
| *CDH2* | *FGFR3* | *MAP2K4* | *PRKDC* | *WT1* |  |  |
| *CDH20* | *FGFR4* | *MCL1* | *PTCH1* |  |  |  |
| *CDH5* | *FLT1* | *MDM2* | *PTCH2* |  |  |  |
| *CDK4* | *FLT3* | *MDM4* | *PTEN* |  |  |  |

**Additional File 1, Table S1: 182 genes sequenced across entire coding sequence (A) and 14 genes sequenced across selected introns (B).**

| Sample ID | HPV status | Median unique exon coverage | Total read-pairs sequenced |
| --- | --- | --- | --- |
| P6 | HPV positive | 1,076 | 48,299,162 |
| P7 | HPV negative | 1,107 | 54,626,192 |
| P8 | HPV positive | 297 | 48,736,330 |
| P10 | HPV negative | 218 | 49,463,347 |
| P12 | HPV negative | 328 | 47,329,276 |
| P13 | HPV positive | 277 | 40,139,612 |
| P14 | HPV negative | 414 | 47,958,763 |
| P17 | HPV negative | 888 | 57,647,562 |
| P19 | HPV positive | 748 | 55,176,974 |
| P24 | HPV negative | 276 | 48,438,015 |
| P25 | HPV negative | 463 | 44,939,294 |
| P26 | HPV positive | 860 | 45,939,532 |
| P28 | HPV positive | 131 | 47,599,531 |
| P29 | HPV negative | 555 | 47,788,462 |
| P35 | HPV positive | 1,134 | 63,082,397 |
| P38 | HPV positive | 166 | 75,032,374 |
| P40 | HPV negative | 826 | 49,462,948 |
| P43 | HPV positive | 969 | 42,999,459 |
| P50 | HPV positive | 486 | 60,363,994 |
| P60 | HPV positive | 292 | 54,457,951 |
| P62 | HPV negative | 127 | 48,397,631 |
| P67 | HPV positive | 1,099 | 51,745,349 |
| P70 | HPV negative | 437 | 56,976,678 |
| P72 | HPV positive | 695 | 38,092,695 |
| P74 | HPV positive | 162 | 32,269,824 |
| P79 | HPV positive | 295 | 15,322,671 |
| P82 | HPV positive | 654 | 26,001,115 |
| P83 | HPV positive | 339 | 54,070,149 |
| P90 | HPV negative | 382 | 57,871,085 |
| P91 | HPV negative | 104 | 46,936,604 |
| P92 | HPV negative | 99 | 43,687,441 |
| P94 | HPV negative | 98 | 51,275,877 |
| P95 | HPV negative | 229 | 43,439,472 |
| P105 | HPV positive | 1,525 | 46,694,812 |

**Additional File 1, Table S2: Summary of sequencing details for study samples.**

|  | Gene | Amino acid change | Nucleotide change | Genome position | Sequence coverage | Percent reads |
| --- | --- | --- | --- | --- | --- | --- |
| P6 | FBXW7 | R505H | 1514G>A | chr4:153247288 | 2429 | 8 |
|  | FBXW7 | R505C | 1513C>T | chr4:153247289 | 2417 | 12 |
|  | FBXW7 | R479Q | 1436G>A | chr4:153247366 | 1868 | 10 |
|  | PIK3CA | E542K | 1624G>A | chr3:178936082 | 3416 | 26 |
|  | STK11 | P324L | 971C>T | chr19:1223034 | 711 | 3 |
|  | TP53 | R290C | 868C>T | chr17:7577070 | 762 | 6 |
| P7 | PIK3CA | N345K | 1035T>A | chr3:178921553 | 1356 | 38 |
|  | TP53 | R175H | 524G>A | chr17:7578406 | 535 | 27 |
| P8 | FBXW7 | R479Q | 1436G>A | chr4:153247366 | 630 | 41 |
| P10 | TP53 | Y234H | 700T>C | chr17:7577581 | 220 | 16 |
| P12 | PKHD1 | R1081C | 3241C>T | chr6:51897951 | 406 | 62 |
|  | TP53 | Y220S | 659A>C | chr17:7578190 | 420 | 84 |
| P13 | FBXW7 | R505C | 1513C>T | chr4:153247289 | 402 | 35 |
| P14 | TP53 | R273L | 818G>T | chr17:7577120 | 390 | 65 |
| P17 | PIK3CA | E545K | 1633G>A | chr3:178936091 | 1699 | 28 |
|  | TP53 | G154fs*16 | 459_459delG | chr17:7578470 | 578 | 47 |
|  | TET2 | Q1445* | 4333C>T | chr4:106193871 | 984 | 4 |
| P19 | PIK3CA | E542K | 1624G>A | chr3:178936082 | 929 | 8 |
| P24 | TP53 | L130fs*17 | 388_392delCTCAA | chr17:7578537 | 185 | 70 |
| P25 | TP53 | Q165* | 493C>T | chr17:7578437 | 617 | 69 |
| P26 | - | - | - | - | - | - |
| P28 | - | - | - | - | - | - |
| P29 | FGFR3 | S249C | 746C>G | chr4:1803568 | 550 | 15 |
|  | TP53 | Y236* | 708C>A | chr17:7577573 | 294 | 64 |
| P35 | - | - | - | - | - | - |
| P38 | - | - | - | - | - | - |
| P40 | TP53 | R306* | 916C>T | chr17:7577022 | 710 | 84 |
|  | PTEN | splice | 79+1G>T | chr10:89624306 | 787 | 81 |
| P43 | - | - | - | - | - | - |
| P50 | KRAS | G12D | 35G>A | chr12:25398284 | 513 | 25 |
| P60 | - | - | - | - | - | - |
| P62 | TP53 | Y220C | 659A>G | chr17:7578190 | 112 | 55 |
|  | PIK3CA | D1029H | 3085G>C | chr3:178952030 | 184 | 18 |
|  | CDKN2A | splice | 458-1G>A | chr9:21968242 | 91 | 46 |
| P67 | - | - | - | - | - | - |
| P70 | TP53 | Q104* | 310C>T | chr17:7579377 | 480 | 67 |
|  | LRP1B | I2387fs*2 | 7159_7160insA | chr2:141356234 | 374 | 16 |
|  | NOTCH1 | splice | 5019-1G>A | chr9:139397783 | 519 | 71 |
|  | SUFU | G11fs*85 | 30_30delC | chr10:104263938 | 932 | 31 |
| P72 | PIK3CA | E545K | 1633G>A | chr3:178936091 | 1853 | 1 |
| P74 | - | - | - | - | - | - |
| P79 | PTEN | Q245* | 733C>T | chr10:89717708 | 391 | 38 |
| P82 | PIK3CA | H1047L | 3140A>T | chr3:178952085 | 695 | 24 |
| P83 | RB1 | R579* | 1735C>T | chr13:49027168 | 288 | 41 |
| P90 | TP53 | L114fs*35 | 341_342insT | chr17:7579345 | 326 | 57 |
| P91 | TP53 | R337L | 1010G>T | chr17:7574017 | 127 | 29 |
|  | TP53 | L330fs*15 | 986_986delC | chr17:7576859 | 207 | 17 |
| P92 | TP53 | R335L | 1004G>T | chr17:7574023 | 92 | 74 |
|  | TP53 | G334V | 1001G>T | chr17:7574026 | 95 | 73 |
| P94 | TP53 | T155P | 463A>C | chr17:7578467 | 121 | 62 |
| P95 | TP53 | splice | 920-1G>A | chr17:7576927 | 225 | 43 |
| P105 | FBXW7 | R367* | 1099C>T | chr4:153251907 | 1380 | 22 |
|  | KRAS | G12D | 35G>A | chr12:25398284 | 1892 | 25 |
|  | PIK3CA | E545K | 1633G>A | chr3:178936091 | 2760 | 32 |

**Additional File 1, Table S3: Summary of known and likely somatic or functional base substitution and indel (short-variant) alterations detected by deep sequencing.**

| Sample ID | Gene | Amino acid change | Nucleotide change | Genome position | Sequence coverage | Percent reads |
| --- | --- | --- | --- | --- | --- | --- |
| P6 | BRCA2 | S326R | 978C>A | chr13:32906593 | 1034 | 54 |
|  | MET | V13M | 37G>A | chr7:116339175 | 1371 | 8 |
|  | SMARCA4 | R359Q | 1076G>A | chr19:11098558 | 705 | 62 |
|  | STAT3 | G388R | 1162G>C | chr17:40481643 | 1550 | 44 |
| P7 | DNMT3A | A116G | 347C>G | chr2:25505411 | 412 | 47 |
|  | EPHA6 | M635R | 1904T>G | chr3:97194205 | 1310 | 44 |
|  | IGF2R | P722L | 2165C>T | chr6:160468304 | 1168 | 47 |
|  | RET | L56M | 166C>A | chr10:43595999 | 947 | 51 |
|  | RUNX1 | G367S | 1099G>A | chr21:36164776 | 1617 | 12 |
| P8 | FANCA | H292D | 874C>G | chr16:89865593 | 147 | 15 |
|  | FGFR2 | K659N | 1977G>C | chr10:123247514 | 568 | 45 |
|  | PTPRD | G203R | 607G>A | chr9:8524997 | 246 | 40 |
|  | RPTOR | R532Q | 1595G>A | chr17:78857229 | 256 | 50 |
| P10 | ARID1A | E1779G | 5336A>G | chr1:27105725 | 185 | 45 |
|  | BCL6 | D210N | 628G>A | chr3:187447565 | 163 | 50 |
|  | INHBA | R229Q | 686G>A | chr7:41729843 | 175 | 45 |
|  | MRE11A | R604H | 1811G>A | chr11:94179032 | 229 | 39 |
| P12 | AR | R599K | 1796G>A | chrX:66905879 | 214 | 38 |
|  | AURKA | S89C | 266C>G | chr20:54961366 | 974 | 11 |
|  | PDGFRA | S268T | 802T>A | chr4:55133498 | 281 | 65 |
| P13 | AR | N849fs*32 | 2540_2541insA | chrX:66942759 | 316 | 38 |
|  | IGF1R | P190S | 568C>T | chr15:99251264 | 276 | 45 |
|  | PDGFRB | T294A | 880A>G | chr5:149513203 | 237 | 46 |
|  | TET2 | P472L | 1415C>T | chr4:106156514 | 296 | 45 |
| P14 | ABL2 | D46H | 136g>C | chr1:179102486 | 525 | 54 |
|  | EPHA3 | T153I | 458C>T | chr3:89259314 | 626 | 22 |
|  | MUTYH | G23R | 67G>A | chr1:45800153 | 424 | 22 |
|  | PIK3CA | L339V | 1015C>G | chr3:178921533 | 943 | 5 |
| P17 | CHEK2 | E109Q | 325g>C | chr22:29126531 | 710 | 18 |
|  | HRAS | splice | 288_290+150  del153 | chr11:533615 | 878 | 21 |
|  | KRAS | M111V | 331A>G | chr12:25378667 | 2041 | 14 |
|  | LRP1B | N4559S | 13676A>G | chr2:140990879 | 1040 | 17 |
|  | LRP1B | D3337N | 10009G>A | chr2:141208185 | 842 | 22 |
|  | TSC2 | S1466L | 4397C>T | chr16:2134620 | 673 | 51 |

| P19 | ABL2 | T769S | 2305A>T | chr1:179078052 | 682 | 51 |
| --- | --- | --- | --- | --- | --- | --- |
|  | ERBB2 | E1114K | 3340G>A | chr17:37883728 | 682 | 9 |
|  | JAK1 | S383G | 1147A>G | chr1:65330499 | 1110 | 46 |
|  | KDR | I915T | 2744T>C | chr4:55961817 | 686 | 16 |
|  | NTRK2 | E371K | 1111G>A | chr9:87342826 | 1108 | 8 |
| P24 | JAK2 | E900Q | 2698G>C | chr9:5089800 | 247 | 14 |
|  | PKHD1 | I3658T | 10973T>C | chr6:51523951 | 289 | 34 |
| P25 | FLT1 | D399E | 1197T>G | chr13:29001968 | 449 | 67 |
|  | FLT3 | I542M | 1626C>G | chr13:28608516 | 376 | 29 |
|  | MDM2 | D375N | 1123G>A | chr12:69233258 | 643 | 14 |
|  | NOTCH1 | E1341K | 4021G>A | chr9:139400327 | 188 | 73 |
|  | VHL | V13G | 38T>G | chr3:10183569 | 404 | 20 |
| P26 | NOTCH1 | R1438H | 4313G>A | chr9:139400035 | 561 | 54 |
|  | PTCH1 | T1052M | 3155C>T | chr9:98220308 | 703 | 50 |
| P28 | ABL1 | N350D | 1048A>G | chr9:133748330 | 105 | 50 |
|  | BRAF | E24D | 72G>C | chr7:140624432 | 132 | 45 |
|  | BRCA1 | A622V | 1865C>T | chr17:41245683 | 164 | 38 |
|  | CDH2 | G473E | 1418G>A | chr18:25570241 | 122 | 35 |
|  | EPHA7 | E712Q | 2134G>C | chr6:93967218 | 145 | 40 |
|  | FGFR4 | V10G | 29T>G | chr5:176516632 | 111 | 50 |
|  | MLL | G3097fs*2 | 9290_9290delG | chr11:118375905 | 148 | 15 |
|  | PAK3 | D175E | 525t>A | chrX:110406199 | 135 | 12 |
| P29 | EPHA7 | splice | 2173-1G>A | chr6:93965756 | 686 | 9 |
|  | LRP1B | P188T | 562C>A | chr2:142004825 | 520 | 6 |
| P35 | RUNX1 | R250H | 749G>A | chr21:36206763 | 653 | 45 |
|  | ARID1A | E1779G | 5336A>G | chr1:27105725 | 1264 | 48 |
|  | CDH5 | K768Q | 2302A>C | chr16:66437019 | 817 | 51 |
|  | HSP90AA1 | K396del | 1186_1188delAAG | chr14:102551176 | 2010 | 16 |
| P38 | ATM | R2161H | 6482G>A | chr11:108192057 | 129 | 11 |
|  | GPR124 | E150K | 448G>A | chr8:37686816 | 130 | 51 |
|  | MET | D1117N | 3349G>A | chr7:116418838 | 180 | 17 |
|  | NOTCH1 | C1133F | 3398G>T | chr9:139402519 | 133 | 42 |
| P40 | AKT1 | D44fs*19 | 131_131delA | chr14:105246468 | 1054 | 40 |
|  | HOXA3 | P268R | 803C>G | chr7:27148063 | 734 | 48 |
|  | NOTCH1 | D469G | 1406A>G | chr9:139412239 | 1145 | 60 |
|  | NTRK2 | E780D | 2340G>C | chr9:87636175 | 311 | 42 |
|  | SMAD4 | V407L | 1219G>C | chr18:48593468 | 781 | 35 |

| P43 | ATM | N1356D | 4066A>G | chr11:108158399 | 990 | 40 |
| --- | --- | --- | --- | --- | --- | --- |
|  | EPHA3 | L10F | 28C>T | chr3:89156926 | 369 | 38 |
|  | MAP2K2 | P329S | 985C>T | chr19:4095447 | 540 | 49 |
|  | MSH2 | I633T | 1898T>C | chr2:47702302 | 1223 | 52 |
|  | PRKDC | R1735Q | 5204G>A | chr8:48792083 | 554 | 52 |
| P50 | IGF2R | E508fs*48 | 1520_1524 CGGAA>G | chr6:160464219 | 1033 | 18 |
| P60 | EPHB6 | V737M | 2209G>A | chr7:142566420 | 143 | 46 |
| P62 | ARID1A | P1621T | 4861C>A | chr1:27101579 | 87 | 41 |
|  | IRS2 | A701_V702insA | 2103_2104insGCC | chr13:110436297 | 185 | 33 |
|  | LRP1B | G935R | 2803G>A | chr2:141739813 | 147 | 52 |
|  | PRKDC | L1707Q | 5120T>A | chr8:48792167 | 141 | 48 |
|  | TBX22 | E111A | 332A>C | chrX:79278715 | 101 | 16 |
| P67 | BRCA1 | L246V | 736T>G | chr17:41246812 | 1351 | 48 |
|  | RPTOR | R1154C | 3460C>T | chr17:78931513 | 236 | 64 |
| P70 | TET2 | P1962L | 5885C>T | chr4:106197552 | 292 | 24 |
|  | ABL1 | H704R | 2111A>G | chr9:133759731 | 697 | 24 |
|  | BRCA2 | K1314E | 3940A>G | chr13:32912432 | 429 | 9 |
|  | CDH5 | R402M | 1205G>T | chr16:66426274 | 369 | 17 |
|  | CDKN2B | V98L | 292G>C | chr9:22006111 | 1233 | 34 |
|  | ERBB4 | S40fs*1 | 111_112delCT | chr2:212989598 | 316 | 11 |
|  | FGFR4 | P528fs*53 | 1583_1583delC | chr5:176522605 | 633 | 14 |
|  | LRP1B | H2874N | 8620C>A | chr2:141260574 | 462 | 16 |
|  | MLH1 | G67R | 199G>A | chr3:37038192 | 418 | 66 |
|  | NF2 | L290R | 869T>G | chr22:30061037 | 440 | 34 |
|  | NKX2-1 | K181E | 541A>G | chr14:36987058 | 465 | 54 |
|  | NTRK1 | E562K | 1684G>A | chr1:156846243 | 477 | 36 |
|  | TNKS2 | N622fs*29 | 1857_1857delA | chr10:93601945 | 247 | 26 |
|  | USP9X | L28fs*51 | 82_82delC | chrX:40982962 | 435 | 14 |
| P72 | EPHA7 | Y399* | 1197T>G | chr6:94066562 | 647 | 28 |
|  | EPHB6 | E867K | 2599G>A | chr7:142567711 | 297 | 36 |
|  | MITF | T444R | 1331C>G | chr3:70014170 | 948 | 34 |
| P74 | BRCA1 | L246V | 736T>G | chr17:41246812 | 237 | 38 |
|  | RPTOR | R1154C | 3460C>T | chr17:78931513 | 128 | 84 |
|  | TET2 | Q1542_R1543  insQ | 4626_4627insCAG | chr4:106196293 | 184 | 30 |

| P79 | CDH5 | V359I | 1075G>A | chr16:66426144 | 204 | 33 |
| --- | --- | --- | --- | --- | --- | --- |
|  | PIK3CA | H510N | 1528C>A | chr3:178928342 | 582 | 63 |
|  | PRKDC | N3605del | 10813_10815  delAAC | chr8:48701553 | 591 | 18 |
|  | STAT3 | E523K | 1567G>A | chr17:40476762 | 399 | 11 |
|  | TSC1 | H732Y | 2194C>T | chr9:135779052 | 258 | 56 |
| P82 | APC | S2544L | 7631C>T | chr5:112178922 | 741 | 15 |
|  | EPHB6 | G182E | 545G>A | chr7:142562103 | 1112 | 16 |
|  | ERG | P116R | 347C>G | chr21:39795373 | 714 | 50 |
|  | GNAS | Q96E | 286C>G | chr20:57428793 | 432 | 46 |
|  | KIT | T488M | 1463C>T | chr4:55592139 | 719 | 8 |
|  | PHLPP2 | S1194F | 3581C>T | chr16:71683184 | 594 | 15 |
|  | PIK3CG | S236L | 707C>T | chr7:106508713 | 885 | 6 |
|  | SUFU | P482L | 1445C>T | chr10:104389902 | 713 | 48 |
|  | TNKS | M1311I | 3933G>A | chr8:9634195 | 498 | 19 |
| P83 | BRCA2 | L709V | 2125C>G | chr13:32910617 | 255 | 44 |
|  | ESR1 | K299R | 896A>G | chr6:152265443 | 352 | 39 |
|  | HSP90AA1 | S10A | 28T>G | chr14:102605714 | 89 | 52 |
|  | MET | R1004* | 3010C>T | chr7:116412025 | 392 | 18 |
|  | TSC2 | A583T | 1747G>A | chr16:2120487 | 285 | 51 |
| P90 | EPHB4 | P983L | 2948C>T | chr7:100401099 | 204 | 50 |
|  | FBXW7 | V424G | 1271T>G | chr4:153249507 | 275 | 56 |
|  | SMAD3 | K53R | 158A>G | chr15:67358650 | 819 | 37 |
| P91 | CDK4 | V260E | 779T>A | chr12:58143005 | 135 | 45 |
|  | EPHA6 | Q581* | 1741C>T | chr3:97167421 | 124 | 10 |
|  | EPHB6 | S172_S173>S | 516_518delCTC | chr7:142562073 | 162 | 14 |
|  | IGF2R | R1466Q | 4397G>A | chr6:160491044 | 125 | 58 |
|  | JAK2 | E177* | 529G>T | chr9:5050746 | 108 | 14 |
|  | SMARCA4 | E990K | 2968G>A | chr19:11134302 | 102 | 11 |

| P92 | ABL2 | G1143S | 3427G>A | chr1:179076930 | 166 | 60 |
| --- | --- | --- | --- | --- | --- | --- |
|  | ALK | A17V | 50C>T | chr2:30143476 | 125 | 46 |
|  | BRCA2 | I1977L | 5929A>C | chr13:32914421 | 97 | 28 |
|  | CD79A | T140N | 419C>A | chr19:42383644 | 104 | 48 |
|  | FGFR3 | Y337F | 1010A>T | chr4:1805498 | 92 | 66 |
|  | IRS2 | D1068N | 3202G>A | chr13:110435199 | 304 | 51 |
|  | LTK | E752* | 2254G>T | chr15:41796352 | 100 | 50 |
|  | MSH2 | D487E | 1461C>G | chr2:47690244 | 66 | 59 |
|  | NF1 | S637I | 1910G>T | chr17:29552177 | 79 | 18 |
|  | NOTCH1 | T1996S | 5986A>T | chr9:139393660 | 235 | 40 |
|  | PIK3CG | W106* | 317G>A | chr7:106508323 | 170 | 41 |
|  | TOP1 | W754S | 2261G>C | chr20:39751900 | 163 | 29 |
| P94 | DNMT3A | E199D | 357G>C | chr2:25505401 | 49 | 43 |
|  | MSH6 | L1264V | 3790C>G | chr2:48033486 | 95 | 31 |
| P95 | ABL2 | P650T | 1948C>A | chr1:179078409 | 225 | 48 |
|  | DDR2 | C177F | 530G>T | chr1:162725058 | 173 | 15 |
|  | IKBKE | S664L | 1991C>T | chr1:206666657 | 268 | 10 |
|  | PKHD1 | Q1923L | 5768A>T | chr6:51824808 | 271 | 42 |
|  | PTPN11 | T553M | 1658C>T | chr12:112940006 | 261 | 38 |
|  | TSC2 | A583T | 1747G>A | chr16:2120487 | 373 | 40 |
| P105 | LRP1B | N2501D | 7501A>G | chr2:141298554 | 1409 | 6 |
|  | MYCN | E185K | 553G>A | chr2:16082739 | 222 | 19 |
|  | PRKDC | S1512N | 4535G>A | chr8:48800145 | 2314 | 51 |
|  | PRKDC | V1434I | 4300G>A | chr8:48801192 | 1410 | 48 |
|  | SMARCA4 | R906C | 2716C>T | chr19:11132500 | 1208 | 27 |

**Additional File 1, Table S4: Summary of base substitution and indel (short-variant) alterations of unknown status detected by deep sequencing.**

| Sample ID | Gene | Tumor copy number | Amplification/loss |
| --- | --- | --- | --- |
| P6 | SOX2 | 8 | amplification |
|  | PIK3CA | 8 | amplification |
| P7 | CDKN2A | 0 | loss |
|  | CDKN2B | 0 | loss |
| P8 | - | - | - |
| P10 | - | - | - |
| P12 | CCND1 | 12 | amplification |
|  | MDM2 | 12 | amplification |
|  | CDKN2A | 0 | loss |
| P13 | PTEN | 0 | loss |
| P14 | CCND1 | 14 | amplification |
|  | CCND3 | 15 | amplification |
|  | MCL1 | 13 | amplification |
|  | PIK3CA | 8 | amplification |
|  | SOX2 | 8 | amplification |
|  | MYC | 7 | amplification |
|  | CDKN2A | 0 | loss |
|  | CDKN2B | 0 | loss |
| P17 | CCND1 | 16 | amplification |
|  | CDKN2A | 0 | loss |
|  | CDKN2B | 0 | loss |
| P19 | - | - | - |
| P24 | PIK3CA | 8 | amplification |
|  | RICTOR | 8 | amplification |
|  | CDKN2A | 0 | loss |
|  | CDKN2B | 0 | loss |
| P25 | CCND1 | 16 | amplification |
|  | MYC | 8 | amplification |
|  | CDKN2A | 0 | loss |
|  | CDKN2B | 0 | loss |
| P26 | - | - | - |
| P28 | - | - | - |
| P29 | FGFR1 | 10 | amplification |
|  | KDM6A | 0 | loss |
| P35 | PTEN | 0 | loss |
| P38 | - | - | - |
| P40 | CCND1 | 8 | amplification |
|  | CDKN2A | 0 | loss |
|  | CDKN2B | 0 | loss |
|  | FBXW7 | 0 | loss |
| P43 | - | - | - |
| P50 | - | - | - |
| P60 | PTEN | 0 | loss |
| P62 | CCND1 | 16 | amplification |
| P67 | BCL2L1 | 14 | amplification |
|  | SOX2 | 9 | amplification |
|  | PIK3CA | 9 | amplification |
| P70 | - | - | - |
| P72 | PIK3CA | 6 | amplification |
|  | SOX2 | 6 | amplification |
| P74 | BCL2L1 | 11 | amplification |
| P79 | - | - | - |
| P82 | - | - | - |
| P83 | - | - | - |
| P90 | EGFR | 7 | amplification |
|  | CDKN2A | 0 | loss |
|  | CDKN2B | 0 | loss |
| P91 | CCND1 | 13 | amplification |
| P92 | CCND1 | 7 | amplification |
| P94 | RICTOR | 11 | amplification |
| P95 | EGFR | 16 | amplification |
|  | CCND1 | 13 | amplification |
| P105 | - | - | - |

**Additional File 1, Table S5: Summary of copy number alterations detected by deep sequencing.**

**Additional File 1, Table S6: List of *TP53* mutations revealed by deep sequencing in HPV+ and HPV- HNSCC samples.**
