# Supplementary material for: Targeted next-generation sequencing of head and neck squamous cell carcinoma identifies novel genetic alterations in HPV+ and HPV- tumors
Source: Genome Med. 2013 May 29;5(5):49. doi: 10.1186/gm453 (PMC4064312; doi:10.1186/gm453)
Supplement: Additional file 2 — Figure S1: Infinium CNA profiling of HPV+ and HPV- HNSCC samples. Obtained genome-wide copy number alteration profiles (cumulative frequencies) between the two groups are illustrated and chromosomes displaying similar patterns of gain and loss in HPV+ and HPV- HNSCC samples are boxed. Chromosome 6 (MHC regions) and Y chromosome are not shown. Figure S2: Validation of detected mutations by SequenomOncoCarta panels v1.0 and v3.0. Mutations in HNSCC samples detected by deep sequencing were validated using the OncoCartapanels v1.0 and v3.0. 8 out of 9 mutations that were successfully tested on the Oncocarta panel were confirmed (green: confirmed, pink: not confirmed, grey: n/a). *The PIK3CA_E545K mutation in sample P72_pos was called at 1% allele frequency by NGS, and this mutation was therefore unlikely to be detected by Sequenom analysis. [file gm453-S2.DOCX]

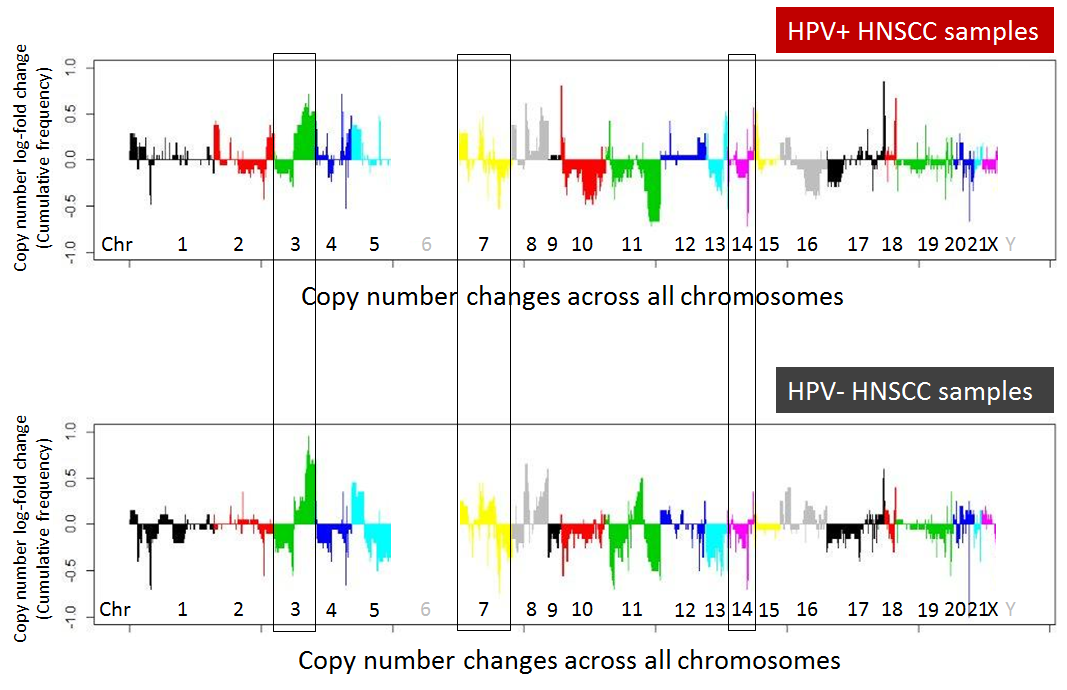


**Additional File 2, Figure S1: Infinium CNA profiling of HPV+ and HPV- HNSCC samples.** Obtained genome-wide copy number alteration profiles (cumulative frequencies) between the two groups are illustrated and chromosomes displaying similar patterns of gain and loss in HPV+ and HPV- HNSCC samples are boxed. Chromosome 6 (MHC regions) and Y chromosome are not shown.

**Supplemental note:**

For chromosomes harbouring concordant copy number changes in both HPV+ and HPV- HNSCC samples (in particular in chromosomes 3, 7 and 14) this may point towards common molecular pathways affected in the pathogenesis of both HPV+ and HPV- cancers.

*

**Additional File 2, Figure S2: Validation of detected mutations by Sequenom OncoCarta panels v1.0 and v3.0.**

Mutations in HNSCC samples detected by deep sequencing were validated using the OncoCarta panels v1.0 and v3.0. 8 out of 9 mutations that were successfully tested on the Oncocarta panel were confirmed (green: confirmed, pink: not confirmed, grey: n/a). *The *PIK3CA*_E545K mutation in sample P72_pos was called at 1% allele frequency by NGS, and this mutation was therefore unlikely to be detected by Sequenom analysis.

**Supplemental note:**

As our NGS exome sequencing targeted the whole gene sequence, whereas Sequenom OncoCarta panels only target specific mutational hotspots of certain genes, the majority of NGS detected mutations were not included in the Sequenom analysis.
